# Cells as the first data scientists

**DOI:** 10.1098/rsif.2022.0810

**Published:** 2023-02-08

**Authors:** Michael L. Wong, Anirudh Prabhu

**Affiliations:** ^1^ Earth and Planets Laboratory, Carnegie Institution for Science, Washington, DC 20015, USA; ^2^ NHFP Sagan Fellow, NASA Hubble Fellowship Program, Space Telescope Science Institute, Baltimore, MD 21218, USA

**Keywords:** information, informatics, data science, evolution, definitions of life

## Abstract

The concepts that we generally associate with the field of data science are strikingly descriptive of the way that life, in general, processes information about its environment. The ‘information life cycle’, which enumerates the stages of information treatment in data science endeavours, also captures the steps of data collection and handling in biological systems. Similarly, the ‘data–information–knowledge ecosystem’, developed to illuminate the role of informatics in translating raw data into knowledge, can be a framework for understanding how information is constantly being transferred between life and the environment. By placing the principles of data science in a broader biological context, we see the activities of data scientists as the latest development in life's ongoing journey to better understand and predict its environment. Finally, we propose that informatics frameworks can be used to understand the similarities and differences between abiotic complex evolving systems and life.

## 1. Introduction

One of the most enigmatic questions in science continues to be *what is life?* (e.g. [1–4]). Despite numerous attempts to define life, there is no single agreed upon characterization of the living state—or even a consensus on whether one is needed [5–7]. This lack of agreement reveals a major gap in scientific understanding with implications for the search for life elsewhere and the creation of de novo life [8]. Few would argue with the idea that information processing is one of the central pillars of life, but a universal definition of information and how exactly information creates a distinction between life and non-life is far from settled.

Today, information is so prevalent in our lives that we have created new domains of science—e.g. data science and informatics—that are centred upon exploring information's multifaceted nature and how it can be used to reveal trends, patterns and truths about our world. The development of informatics has resulted in heuristic methods that elucidate the role of information in data science endeavours. In this contribution, we illustrate how two of these concepts—namely, the ‘information life cycle’ and the ‘data–information–knowledge ecosystem’—can also be used to describe the ways in which information flows through living systems in general. We propose that an informatics perspective may be a particularly illuminating lens through which to understand the differences between living and non-living systems. In our framework, living systems constitute a subset of complex evolving systems that perform the full information life cycle and are characterized by a rich data–information–knowledge ecosystem.

## 2. The role of information in data science

The advent of data science in recent decades has reshaped science and society alike [9]. In the physical sciences (e.g. astronomy, geochemistry and mineralogy), life sciences (e.g. agricultural science, genomics and public health) and social sciences (e.g. economics, linguistics and political science), nearly every sector of modern life has been affected by the so-called ‘big data revolution’. Concomitantly, we have witnessed the rise of informatics—a field that focuses on understanding the structure, properties and activities of scientific information, rather than just its content [10]. In the digital age, it is easy to see how the human world is critically dependent upon flows of information. But could it be that life has been practicing the principles of data science and informatics ever since its inception?

**Figure 1.**
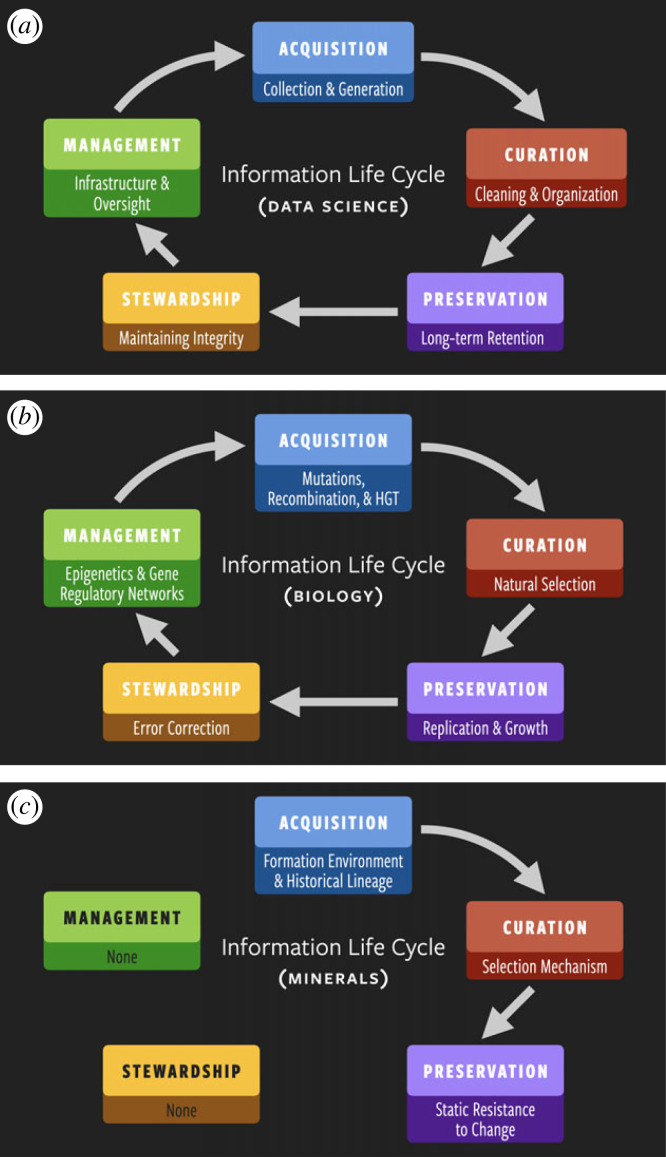
The information life cycle in (*a*) informatics, (*b*) biology and (*c*) minerals. *Inspired by* [10].

Central to data science endeavours is the so-called ‘information life cycle’, which describes the steps that stored information goes through, from its creation to its deletion or archiving (figure 1*a*) [10,11]. The first step is *acquisition*: data must be gathered, perhaps by direct observation or experiment, generated by theory, or rescued from existing but scattered resources. The second step is *curation*: after its collection, data must be cleaned and processed into a state where it is useful; e.g. disparate pieces of information may be standardized and ‘datafied’. The third step is *preservation*: the data must be retained in usable form so that they can be accessed in the future, both for their original purpose and for purposes not yet imagined at the time of collection. The fourth step is *stewardship*: the process of maintaining integrity across acquisition, curation and preservation. The fifth and final step is *management*: creating the infrastructure that oversees all of the other processes, ensuring that previously acquired data are always available to access and use and facilitating further data collection. The functional use of information drives the information life cycle: each step advances the goal of making information more usable, reliable and acquirable.

**Figure 2.**
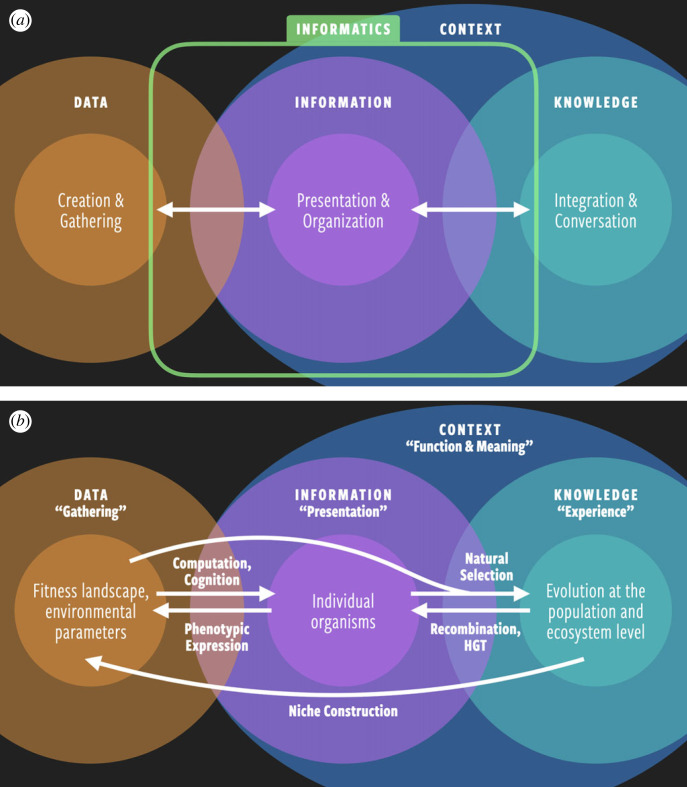
(*a*) The data–information–knowledge ecosystem in data science, highlighting the role of informatics in converting raw data into human knowledge. (*b*) The data–information–knowledge ecosystem in biological systems, cataloguing various transformations and feedbacks between the three domains. In both data science and living systems, information and knowledge are reliant on context. *Inspired by* [10].

Another key concept in informatics is the ‘data–information–knowledge ecosystem’, which describes how data are processed, represented and communicated in a meaningful way (figure 2*a*) [10,11]. Raw *data* are generally useless to the majority of people, so informatics methods must be used to transform data into *information*, which is a representation of the raw data that can be understood by the public. For example, raw data for natural events like hurricanes, earthquakes or volcanoes may include readings from sensors, satellite data and even data from social media posts documenting these natural events. When sensor data or satellite data are plotted into a visualization like a map or a graph, we can finally see the geographical boundaries for the affected areas of a particular hurricane or earthquake.

Once consumers are armed with the appropriate information, they use that information, in combination with other experiences, to gain *knowledge*. For example, meteorologists are able to track the movement of a hurricane and predict the path and intensity of that hurricane based on their expertise and insights they obtained from looking at the information presented to them from a combination of data sources. Knowledge, therefore, is not something acquired in solitude; it is a collective phenomenon reliant on social experiences and context.

The modus operandi of data science is to amass and process large quantities of data in order to draw statistically robust correlations, find previously undiscovered associations or make predictions and/or recommendations that rival or even supersede those of analytical theory. The characterization of complex systems is one arena where data science shines. It is often challenging to derive analytical laws for phenomena with many dynamical components that are influenced by forces at a wide range of spatial and temporal scales. Instead, data science methods can be used to glean accurate and predictive statistical laws. Although big data analytics alone may not be able to divine causality, the discoveries that data science makes can be used to motivate new physical explanations and novel scientific narratives. For example, mineral occurrence data can be used to form mineral association rules that give us the ability to predict previously unknown mineral occurrences and also provide insights into formational environments of minerals and their characteristics [12]. For myriad complex phenomena, data science has helped us reach beyond the limitations of traditional reductionist theory. Will data science approaches eventually lead us to an ultimate description of reality that is superior, in predictive and explanatory power, to compact, ‘physicsy’ descriptions of nature? We leave this question to the future and a discussion of its implications to a more philosophical text.

## 3. Seeing information in life through a data science lens

Information gathering, processing and transference are fundamental aspects of life. Biological systems are learning systems: they record information about their environment and process that information to enhance their ability to survive [13]. Life has evolved manifold ways to perceive its environment, from chemical receptors to vision to magnetoreception. Our mammalian neurological architecture allows us to integrate huge amounts of data to compute our surroundings and make useful predictions about the world—e.g. if I climb a tree, is that lion less likely to eat me?—but even the simplest unicellular life forms (including their ‘dormant’ spores) can perform complex cognitive tasks, like memory and decision-making [14,15]. Indeed, populations of replicating entities can be understood to perform Bayesian inference, and Darwinian evolution can be equated to a search algorithm built upon the basic principles of replication, variation and selection [16–18]. We propose that life does (and has always done) data science–analogue activities without necessarily being ‘conscious’ of it.

The information life cycle of data science is also a valid summary of major informational processes in biological evolution (figure 1*b*). The *acquisition* stage describes the generation of novel genetic sequences, primarily driven by mutations to the germline, recombination and horizontal gene transfer. The *curation* stage is performed by natural selection, pruning a wide range of possible information-bearing states to a smaller number of viable ones. The *preservation* stage is achieved through replication, reproduction and growth, ensuring that genomic information persists and proliferates through time. (We note that in biological systems, curation and preservation are intimately linked: natural selection operates upon reproducing systems. However, it is useful to keep these stages distinct because non-living systems can exhibit selection without replication (e.g. mineral paragenesis) and growth without curation (e.g. wildfire).) The *stewardship* stage is performed by error-correcting mechanisms, such as enzymes that perform kinetic proofreading during DNA replication (e.g. [19–23]). The *management* stage involves a host of mechanisms that control gene expression, such as epigenetic markers (e.g. [24]), gene regulatory networks (e.g. [25]) and factors that tune mutation rate and the uptake of new genes from the environment (e.g. [26,27]).

The modern biological mechanisms responsible for stewardship and management are the result of billions of years of evolution, and it is likely that near the origin of life, steps four and five emerged from the first three steps of the information life cycle. Once evolved, stewardship and management cemented themselves in the information life cycle by benefiting preservation and evolvability—a feedback akin to how stewardship and management in data science facilitate data sharing and collaboration, which in turn help with the acquisition and processing of yet more data.

There is also an analogue to the data–information–knowledge ecosystem present in living systems (figure 2*b*). Here, *data* represent the physical features of the environment—temperature, salinity, chemical gradients, seasonal cycles, random fluctuations, etc. By sensing and computing their environment, biological systems transform data into *information* that is relevant to the survival of an organism or a group of organisms. Examples of the transduction of environmental data into biological information include: how the retina converts photons into nerve impulses [28], how cells transform physical forces into chemical signals [29] and how any number of stimuli can result in modifications to the biochemical circuitry of the proteome [30,31].

In our framework, information is distinct from data in that information contains ‘meaning’ in a given context. Here we draw inspiration from the field of informatics, in which the term ‘data’ refers to raw bits in nature, whereas ‘information’ refers to data that have gained meaning through context or function. In other words, information is a product of data going through the information life cycle; it is data in use. For example, a sequence of nucleobases in a DNA polymer means nothing without the enzymatic machinery required to transcribe and translate it into a polypeptide. In the context of life on Earth, DNA exists within the biological context of a living cell, so it serves a specific function and derives its meaning from its role in the so-called ‘central dogma of molecular biology’ [32]. Should a molecule of DNA exist in some extraterrestrial biosphere whose exobiochemistry has no use for it, that DNA strand would not contain information in our sense of the word; rather, it would merely be a piece of raw data in the environment.

An individual biological unit of selection is essentially a proposal from the biosphere to the environment—a prediction put to the test against new data. Via natural selection over multi-generational time, certain predictions will be strengthened while others are discarded, and populations of individual agents will gain lasting *knowledge* of their environment—recipes of success written in genetic code. Hence, knowledge in the biological sense is like knowledge in the data science sense: it can only be gained through ‘experience’ over time at the communal level.

Biological systems create information from data and knowledge from information (rightward arrows in figure 2*b*), but so too do they create new information from knowledge and new data from information (leftward arrows in figure 2*b*). By existing within the environment with respect to other organisms, and by influencing the environment through phenotypic expression and niche construction, life creates more environmental data and alters the fitness landscape for other life [33–35]. Through recombination (e.g. exercising the ‘knowledge’ of sexual reproduction) and horizontal gene transfer, populations steadily produce new individual variations. Thus, the data–information–knowledge ecosystem is made complete by the ways in which individual organisms and ecosystems impact their environment, changing the data that must be gathered by future generations and compelling the coevolution of life and planet.

Information processing and preservation is an energetically costly undertaking; life's emergence and continual evolution must be driven by the non-negligible complexity—i.e. the high data content—of its environment [36]. Although the emergence of life is still shrouded in mystery, information processing was probably a key feature responsible for the transition between non-living and living systems [37,38]. Numerous proposals have been made for how abiotic systems could have begun transforming environmental data into meaningful information, including but not limited to RNA template-driven replication (e.g. [39]), amyloidal self-propagation (e.g. [40]), mineral templating (e.g. [41,42]) and associative learning in chemical systems [43]. Despite the great diversity of environments and materials proposed to be responsible for the origin of life, in each of these scenarios, information in a proto-living system promotes that system's persistence over time, reflecting the general principle that information processing is a hypothesis-agnostic pillar of life [13].

Life is a data collection and information-processing system built from organic chemistry, powered by free energy gradients and streamlined via natural selection. In our view, life has been honing the core activities of data science for over 3.5 billion years. Over deep time, biology has created genetic knowledge of nearly every kind of environment on Earth (and is now experimenting with living beyond it). As life spread and gained influence over its surroundings, it created new environments and learned how to survive in those too. But life doesn't merely learn; it learns to learn better (e.g. [44–49]). Through a series of evolutionary innovations and major transitions, biology has enhanced its data-gathering and information-processing abilities [50], invented minds that can infer causality [51], produced the dataome [52], expanded its cognitive horizon by orders of magnitude from the microscopic to the planetary [53] and may potentially begin to consciously influence its own evolutionary trajectory [54–56].

Strikingly, many artificial intelligence techniques and computer algorithms are inspired by natural systems [57,58] including: artificial neural networks [59,60], evolutionary algorithms (e.g. genetic algorithms) [61,62], swarm intelligence [63,64], artificial immune systems [65,66], communication networks [67] and even a slime mould-inspired algorithm for mapping the distribution of dark matter across the universe [68]. Just as new discoveries in biology will be enabled by innovations in data science, data science will continue to benefit from a deeper understanding of how biological systems organize and process information and learn from their surroundings.

In this light, the difference between the informatics activities of data scientists and the rest of the living world can be viewed as one not of kind but of degree. Combining the evolutionary gifts of neurological cognition with the technological powers and mathematical techniques of today, human informaticians are engaging in data acquisition, curation, preservation, stewardship and management at unprecedented scale and velocity to wield predictions about nearly every aspect of the known universe with extraordinary precision. But this is hardly a unique endeavour—we humans are just the first to notice the unity of informatics principles at play across the tree of life. The principles of data science are expressed across the expanse of biology, from nanoscopic virions to the largest clonal tree to your friendly AI engineer. To think, it all started with a cell!

## 4. Leveraging an informatics perspective in the quest for a theory of life

Through the lens presented here, the essence of informatics is fundamental to what life is (or, perhaps better put, what life *does*). We propose that atoms, molecules, stars, planets, minerals, hurricanes and other prebiotic or non-living systems do not display the same kind of information life cycle or data–information–knowledge ecosystem as biological systems. Thus, the way that life assimilates data and uses it to enhance its own persistence could be a defining distinction between abiotic and biotic systems. Furthermore, although we take Darwinian evolution as an exemplar of biological information processing over deep time on Earth, the abstract principles of informatics and learning may be more universal frameworks for understanding extraterrestrial life, which may not necessarily use Darwinian evolution to update its knowledge about its surroundings [13,69].

The act of transforming data into a state that increases a system's survivability generates *functional* [70] or *semantic* [71] information. Take, for example, a bacterial gene that produces an enzyme that metabolizes a certain nutrient in the environment. This gene is the result of mutations and/or recombinations that explored the vast combinatorial space of nucleobase strings (acquisition) and was selected for by differential reproductive success (curation and preservation). If this gene can be turned on and off depending on the concentration of the nutrient in the environment, the cell will be spared the expense of producing a needless enzyme when the nutrient is scarce, further enhancing survivability (management). Via the information life cycle, raw data, in the form of fluctuating concentrations of nutrients, have been turned into various layers of functional information, in the form of the genetic, epigenetic and enzymatic apparati that help the cell persist and proliferate.

Life represents a subset of all known complex evolving systems, which are broadly defined as systems where (i) a large number of interacting components results in a potentially large combinatorial space, (ii) one or more mechanisms exist to generate numerous configurations within that combinatorial space, and (iii) a selection mechanism favours certain configurations over others (e.g. [72–78]). While non-biological complex evolving systems contain information, the information life cycle—and hence the degree of functional information within them—is stunted compared with biological systems.

Let us take mineral evolution as a characteristic example. In general, the minerals that form are those that minimize the free energy of the system at the pressure–temperature–composition conditions during crystallization. Hence, data about the paragenetic environment are recorded in the mineral's chemical and isotopic composition, crystal structure, habit and its context within a mineral assemblage (e.g. [79–90]). Furthermore, the increasing diversity of mineral species and natural kinds through time reflects the increasing chemical complexification of the cosmos [91].

However, the data that minerals contain do not participate fully in the information life cycle (figure 1*c*). First, in minerals, preservation is static (resistance to chemical change) rather than a dynamic process (like cellular replication); the identity of a crystal is tied to its physical substrate, whereas the information in an organism will be replicated many times from new material. Second, the steps of stewardship and management are non-existent for minerals. Third, while the information content of mineralogical systems can be updated by subsequent alteration in changing environments, there is no feedback between the information that has already been generated and the acquisition of new data.

In other words, minerals are essentially geological flash drives: they record data imparted on them by external forces—information that can be erased, updated or overwritten with time (e.g. the conversion of graphite to diamond)—but they do not correct defects in acquired data or otherwise use their stored data to ensure the fidelity of the other steps in the information life cycle. These kinds of differences limit the amount of functional information in abiotic systems to a narrow range of modalities (in minerals, to dissipation and static persistence) and prevent them from evolving open-endedly. Perhaps one axis for measuring ‘lifelikeness’ is the degree to which a system performs informatics processes and exhibits a data–information–knowledge ecosystem.

Finally, we wish to emphasize that on a living planet, information flows *between* biotic and abiotic systems (figure 2), blurring the distinction between life and its environment. Roughly half of the mineral species on Earth are biologically mediated [92], and the nature of Earth's atmosphere has been reshaped over evolutionary time through the exchange of metabolic gases (e.g. [93–95]). At the macroscopic scale, the information life cycle that the biosphere engages in will certainly include traditionally non-biological factors. A fuller exploration of this idea is saved for future work.

We contend that understanding how data are acquired and processed into functional information will be instrumental in developing a richer understanding of complex evolving systems, and to building a general theory of life. A full examination of the role that information plays in various biotic and abiotic systems requires a more granular level of exploration than we can cover here. The details of information content and the degree of information processing differ greatly across non-living systems; for instance, how would one weigh the functional information content of a star versus that of a river channel? Future work in information theory is required before we can truly characterize and compare these disparate physical systems with one another—and with life—on an equal footing. With the tools of data science and informatics at our disposal, perhaps the answers to these fundamental questions are finally within reach.

## Data Availability

This article has no additional data.
